# An interpretable framework for inter-observer agreement measurements in TILs scoring on histopathological breast images: A proof-of-principle study

**DOI:** 10.1371/journal.pone.0314450

**Published:** 2024-12-05

**Authors:** Abdulkerim Capar, Dursun Ali Ekinci, Mucahit Ertano, M. Khalid Khan Niazi, Erva Bengu Balaban, Ibrahim Aloglu, Meryem Dogan, Ziyu Su, Fugen Vardar Aker, Metin Nafi Gurcan

**Affiliations:** 1 Center for Artificial Intelligence Research, Wake Forest University School of Medicine, Winston-Salem, North Carolina, United States of America; 2 Informatics Institute, Istanbul Technical University, Istanbul, Turkiye; 3 Department of Pathology, Haydarpasa Numune Education and Research Hospital, University of Health Sciences, Istanbul, Turkiye; Qatar University College of Medicine, QATAR

## Abstract

Breast cancer, a widespread and life-threatening disease, necessitates precise diagnostic tools for improved patient outcomes. Tumor-Infiltrating Lymphocytes (TILs), reflective of the immune response against cancer cells, are pivotal in understanding breast cancer behavior. However, inter-observer variability in TILs scoring methods poses challenges to reliable assessments. This study introduces a novel and interpretable proof-of-principle framework comprising two innovative inter-observer agreement measures. The first method, Boundary-Weighted Fleiss’ Kappa (BWFK), addresses tissue segmentation predictions, focusing on mitigating disagreements along tissue boundaries. BWFK enhances the accuracy of stromal segmentation, providing a nuanced assessment of inter-observer agreement. The second proposed method, the Distance Based Cell Agreement Algorithm (DBCAA), eliminates the need for ground truth annotations in cell detection predictions. This innovative approach offers versatility across histopathological analyses, overcoming data availability challenges. Both methods were applied to assess inter-observer agreement using a clinical image dataset consisting of 25 images of invasive ductal breast carcinoma tissue, each annotated by four pathologists, serving as a proof-of-principle. Experimental investigations demonstrated that the BWFK method yielded gains of up to 32% compared to the standard Fleiss’ Kappa model. Furthermore, a procedure for conducting clinical validations of artificial intelligence (AI) based cell detection methods was elucidated. Thoroughly validated on a clinical dataset, the framework contributes to standardized, reliable, and interpretable inter-observer agreement assessments. This study is the first examination of inter-observer agreements in stromal segmentation and lymphocyte detection for the TILs scoring problem. The study emphasizes the potential impact of these measures in advancing histopathological image analysis, fostering consensus in TILs scoring, and ultimately improving breast cancer diagnostics and treatment planning. The source code and implementation guide for this study are accessible on our GitHub page, and the full clinical dataset is available for academic and research purposes on Kaggle.

## Introduction

Breast cancer remains one of the most prevalent and life-threatening diseases affecting women worldwide [1]. Early and accurate diagnosis and effective treatment strategies are pivotal in improving patient outcomes and survival rates. Tumor Infiltrating Lymphocytes (TILs) are immune cells that infiltrate the tumor microenvironment and are believed to reflect the individual immune response against cancer cells [2]. The quantity and quality of TILs play a significant role in determining the anti-tumor immune response in breast cancer and various other types of tumors. The biological characteristics and response to treatment in breast cancer vary depending on TILs density levels. Breast cancer is an immunogenic tumor, especially triple-negative breast cancer (TNBC) [3]. Different TILs densities can vary the biological characteristics and response to treatment in breast cancer [4]. TILs have shown predictive and prognostic value in determining the response to neoadjuvant chemotherapy, particularly in HER2-positive and TNBC sub-groups. Assessing TILs as a biomarker in breast cancer is crucial for understanding the tumor’s interaction with the immune system. To achieve accurate assessments, standardized methods are essential, and integrating evolving technology can optimize objectivity. Such advancements have been linked to a stronger immune response, which holds significant implications for prognosis and treatment outcomes [2, 4].

The International Immuno-Oncology Biomarker Working Group on Breast Cancer, commonly known as TILs-WG [5], has developed a comprehensive set of guidelines for visual TIL assessment (VTA) on hematoxylin and eosin (H&E)-stained slides [6–10]. Traditionally, TILs scoring has heavily relied on the expertise of pathologists, who visually assess tissue samples under a microscope. However, this manual scoring process has limitations, including high intra and inter-observer variability and time-intensive demands [11–13]. Precision, efficiency, and consistency in the TILs assessment will facilitate the more effective use of this measurement in clinical settings and cancer care.

Our comprehensive literature search noted that inter-observer variabilities have primarily been addressed within the context of TILs scores [12–17]. However, we have not found any studies that systematically evaluate and quantify the variations in how pathologists segment the stroma and detect lymphocytes within the stroma.

In radiology, some studies have explored the measurement of inter-observer variability through segmentation [18–20]. These studies assess the accuracy and agreement between manual and automated segmentation methods used to delineate organs or lesions in radiological images. An inter-observer segmentation variability was evaluated on ultrasound images [18]. The authors proposed an inter-observer reliability study comparing the performance of a deep learning segmentation model against three experts who manually segmented suspicious breast lesions in clinical ultrasound images. To evaluate inter-observer variability, they compared segmentation masks between pairs of observers and computed the Pearson correlation coefficient. Wilcoxon signed rank tests were used to determine if the model’s performance aligns with the experts. The evaluation used the common medical segmentation metrics, the Dice coefficient, sensitivity, specificity, Cohen’s kappa adapted for segmentation and 95% symmetric Hausdorff distance. Armato et al. categorized the lung nodule boundary annotations according to their size (“nodule ≥ 3 mm,” “nodule < 3 mm,” and “non-nodule ≥ 3 mm”) and measured the inter-observer agreements in these categories [20]. In a recent study to evaluate inter-annotator agreement, the authors assessed the inter-annotator reliability in lesion segmentation on cervical images and abnormality segmentation in Chest X-ray (CXR) images [19]. They extended kappa coefficients, particularly Fleiss’ kappa coefficient, from categorical classification to pixel-wise segmentation by generating and interpreting the new kappa tables for the image segmentation problem. They also proposed two agreement heat-maps to visualize and quantify the inter-annotator reliability, including a common and ranking agreement heat-map. While the method they developed can assess inter-observer segmentation agreements, it lacks the ability to effectively reduce the impact of naturally occurring discrepancies along the region boundaries.

Assessing the agreement between different observers (without ground truth) in histopathological cell detection poses a challenge due to variations in the positions of markers on the same cell and the closeness of neighboring cells. Few studies have tackled this problem. Amgad et al. approached this issue in breast tissues by utilizing agglomerative hierarchical clustering of bounding boxes, employing intersection-over-union (IOU) as a similarity measure [21]. They applied a clustering constraint to avoid merging annotations in cases where a single participant had marked overlapping nuclei. Another cell detection agreement study on light-sheet microscopy images was proposed by Lo Vercio et al. [22]. When calculating cell detection agreement, they still needed ground truth information to understand the cell regions on which they would base their assessments. The matching criterion was that a cell in the ground-truth had a corresponding cell in the automatically extracted segmentation if there was more than 50% overlap between the segmented cells. The object-level F-score was used to quantify the agreement in object-level recognition. Han et al. conducted a study on cell segmentation in immunofluorescence multiplexed images, employing a two-stage domain adaptation approach and weakly labeled data [23]. They also examined the inter-observer agreement concerning segmented cell masks and boundary contours. To assess observer compliance for each cell region, they used error metrics such as Object-Dice and Object-Hausdorff distance. However, it’s important to note that they still required ground truth cell annotations to calculate these metrics. In a recent study by Kang et al., the focus was on assessing the level of agreement among cell annotators when analyzing histopathological images [24]. To accomplish this, one of the annotators was chosen as the ’anchor annotator’, and their annotations were used as the reference point for evaluating the consistency of the other annotators’ work. The researchers developed a conformity measurement algorithm for each annotator, which involved comparing their annotated cells with a control set based on the anchor annotator’s annotations. Similar to previous studies, this approach incorporates ground truth information derived from the anchor’s annotations.

To tackle these challenges, we propose an innovative framework and a proof-of-concept study introducing two novel inter-observer agreement measures tailored for tissue segmentation and cell detection. The first method, known as the Boundary-Weighted Fleiss’ Kappa (BWFK), is designed to assess tissue segmentation agreements with a unique focus on mitigating disagreements along tissue boundaries. Acknowledging the inherent difficulty in precisely delineating boundaries, the BWFK method strategically reduces the impact of annotations near regional edges. This nuanced approach aims to eliminate minor disagreements along tissue boundaries, providing a more accurate reflection of inter-observer agreement in stromal segmentation. Importantly, the BWFK method enhances the interpretability of regional segmentation tasks, expanding its potential for broader applications beyond TILs scoring.

The second proposed method, the Distance Based Cell Agreement Algorithm (DBCAA), represents a novel method for measuring cell detection prediction agreements. Notably, DBCAA is the first of its kind, eliminating the need for ground truth annotations in the evaluation of cell detection. This innovation allows for a more versatile and practical application across various histopathological analyses, overcoming limitations posed by the unavailability or difficulty in obtaining ground truth data.

Furthermore, the proposed BWFK and DBCAA methods offer a unique advantage in their seamless adaptability to assess the concordance of artificial intelligence (AI)-based methods with pathologists. As the field of histopathological image analysis increasingly incorporates AI algorithms for segmentation and detection tasks, the need for reliable metrics to measure the agreement between AI predictions and human observations has become paramount. BWFK, with its boundary-weighted approach and DBCAA, which requires no ground truth annotations, presents versatile solutions that can be effortlessly extended to evaluate the performance of AI algorithms alongside pathologists.

To validate the effectiveness of our proposed framework, we conducted a thorough evaluation on a clinical dataset comprising 25 breast tissue images from 11 patients with invasive ductal breast carcinoma. These images were meticulously annotated by four pathologists, and inter-observer measurements were undertaken for stromal segmentation, lymphocyte detection, and derivation of final TILs scores. Given that this process is exceptionally time-consuming and requires careful attention to detail, we made the decision to limit our dataset to 25 images. This ensured that our annotations were thorough and accurate, despite the smaller sample size.

Both BWFK and DBCAA collectively form an interpretable framework that transcends the confines of TILs scoring, providing a comprehensive solution for understanding the high inter-observer variability in breast cancer pathology. The versatility of these measures facilitates their seamless integration into diverse histopathological applications, contributing to the establishment of standardized, reliable, and interpretable inter-observer agreement assessments. In this paper, we detail the development and application of these methods, highlighting their potential impact on advancing the field of histopathological image analysis and fostering greater consensus in TILs scoring.

The contributions of the proposed study to the literature can be summarized as follows:

- This is the first study to examine inter-observer agreements regarding scores, specifically in stromal segmentation and lymphocyte detection for the TILs scoring problem.
- We introduce a novel inter-observer agreement measure for tissue segmentation predictions called BWFK that is designed to address disagreements along tissue boundaries, providing a more nuanced assessment of inter-observer agreement.
- We propose a novel DBCAA that does not need ground truth annotations to measure inter-observer agreement.
- A pilot study also demonstrated how the clinical validation of AI models can be performed using the DBCAA and BWFK methods.

## Methods

**Fig 1:**
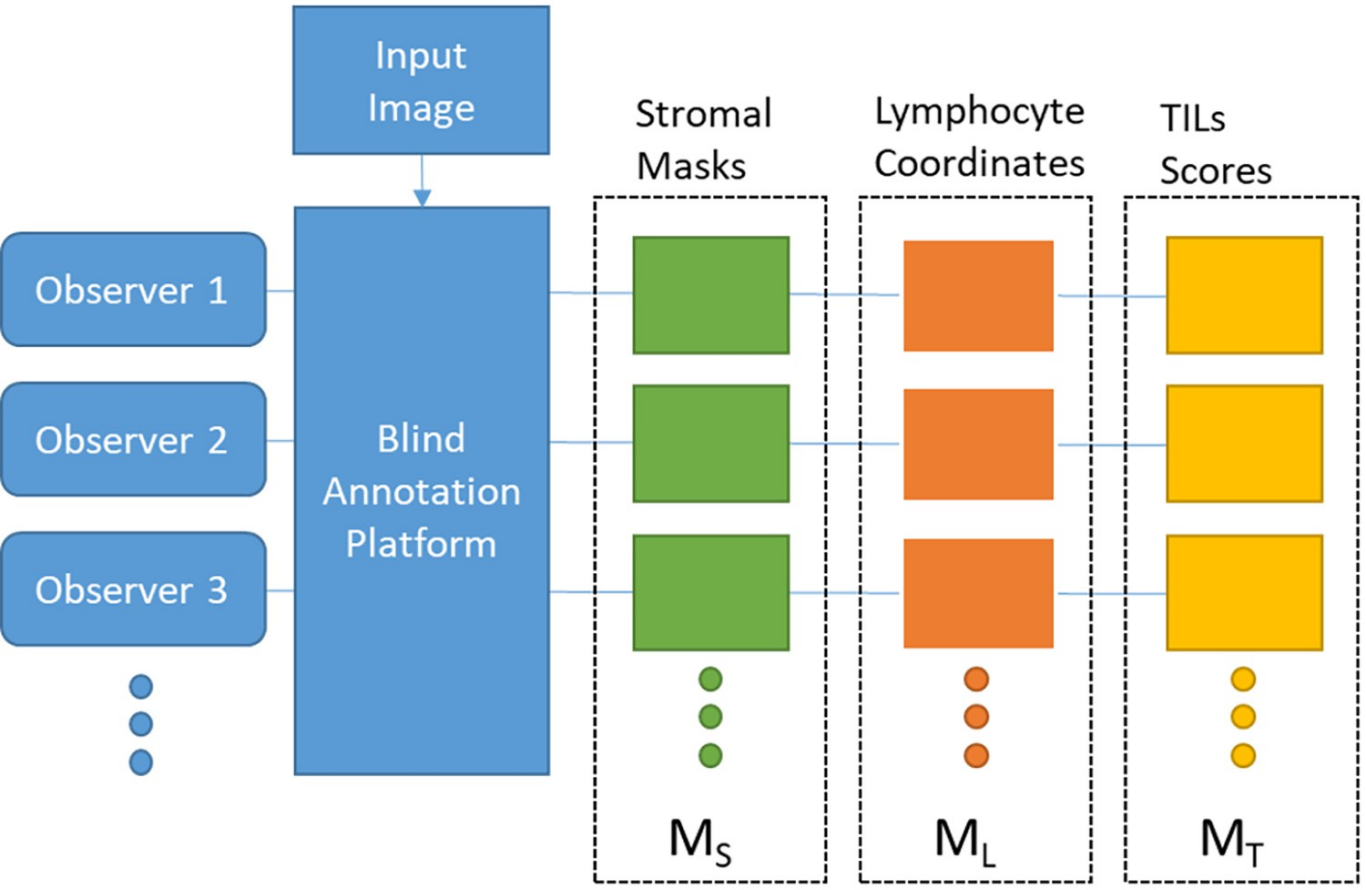
Schematic view of the proposed inter-observer agreement measurement framework. The chart illustrates the workflow for an input image to obtain M_S_, M_L_ and M_T_ statistics from the observers’ annotations employing the Blind Annotation Platform (explained at the end of Methods section).

We categorize the inter-observer agreement measures into stromal agreement (M_S_), lymphocyte agreement (M_L_), and TILs score agreement (M_T_), as depicted in Fig 1.

### Region-based agreement for stroma (M_S_)

The commonly employed evaluation of inter-observer agreements in regional segmentation typically includes pair-wise assessments using Intersection over Union (IoU)-based metrics. However, these metrics are inadequate for evaluating collective agreements involving more than two observers. To address this limitation, we introduce a novel method, the BWFK measure, designed to assess collective inter-observer agreements.

#### Fleiss’ Kappa (FK) measure

FK is a statistical metric used to evaluate agreement reliability among a specific group of observers when assigning categorical ratings to items (subjects) or classifying them. It represents an improved version of Cohen’s Kappa, which assesses agreement scores between two observers. General Kappa score is defined as

$$\kappa =\frac{\bar{P}-\bar{P_{e}}}{1-\bar{P_{e}}}$$ (1)

where $\bar{P}$ is the proportion of observed agreements and ${\bar{P}}_{e}$ is the proportion of agreements expected by chance. Let the subjects be indexed by i = 1,…,M, and the categories be indexed by j = 1,…K. Let N be the number of observers per subject and n_ij_ is the number of observers who annotate the i^th^ subject to the j^th^ category. $\bar{P}$ and ${\bar{P}}_{e}$ values can be calculated as.


$$\bar{P}=\frac{1}{N}\sum_{i=1}^{N}P_{i}=\frac{1}{MN(N-1)}\left(\sum_{i=1}^{M}\sum_{j=1}^{K}n_{ij}^{2}-MN\right)$$ (2)

$${\bar{P}}_{e}=\sum_{j=1}^{N}p_{j}^{2},\;p_{j}=\frac{1}{MN}\left(\sum_{i=1}^{M}n_{ij}\right)$$ (3)

FK statistics can be adapted to image segmentation-based agreement measurements as treating individual pixels within segmentation mask images as subjects [19]. In order to evaluate inter-observer agreements in stromal segmentation the categories were adapted as stroma and non-stroma (K = 2). An example agreement count table is shown in Table 1 where n_ij_ represents the number of observers who annotated the i^th^ pixel to the j^th^ category of stroma (1) or non-stroma (0) categories.

**Table 1:** Counts of agreement between observers on binary segmentation of stroma.

	Category	
Pixel	Non-stroma (0)	Stroma (1)
1	n_10_	n_11_
2	n_20_	n_21_
3	n_30_	n_31_
…	…	…
M	n_M0_	n_M1_

### Boundary-Weighted Fleiss’ Kappa (BWFK) measure

**Fig 2:**
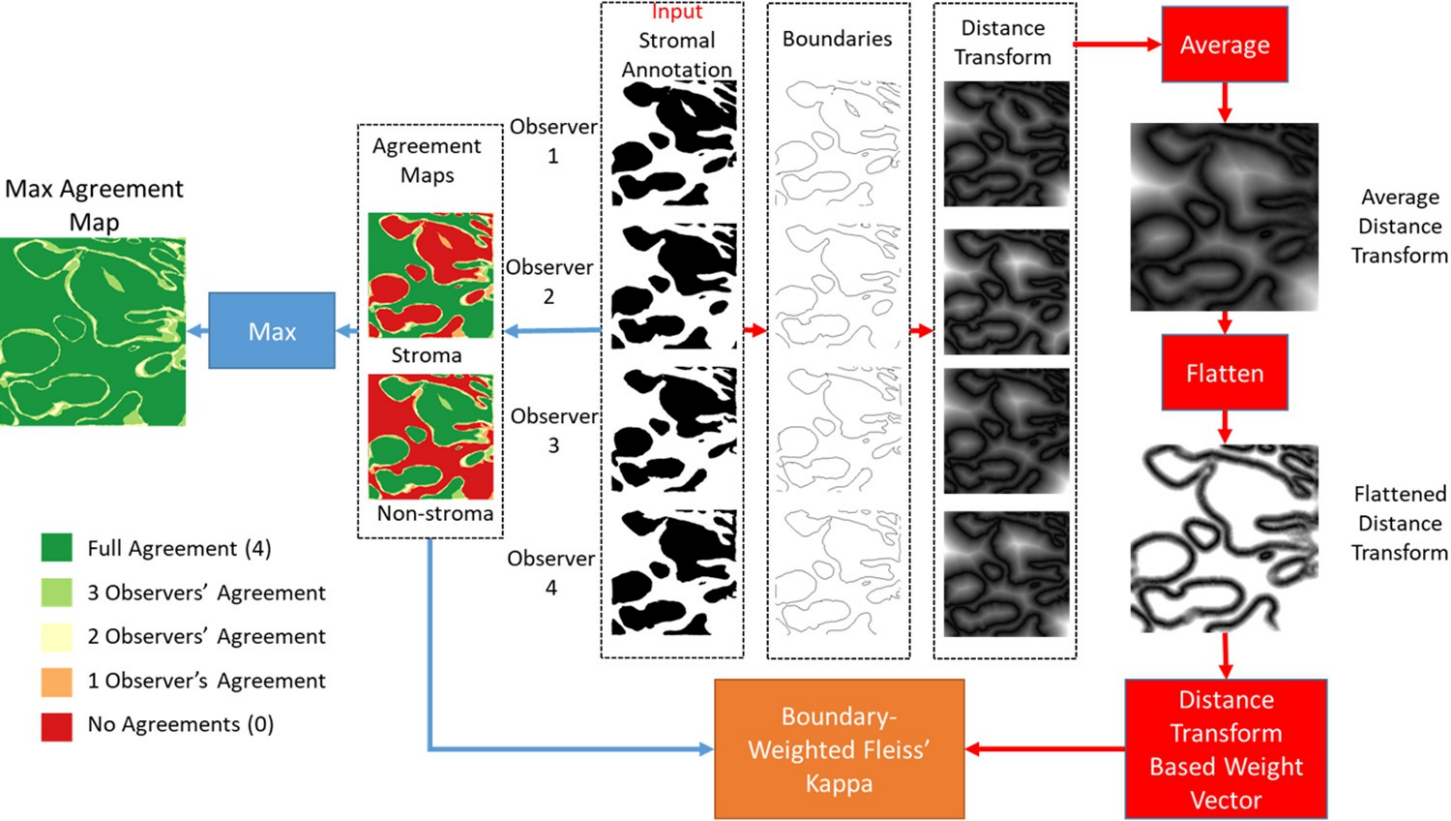
The workflow of the proposed BWFK measurement method for four observers is illustrated. The data processing flows for agreement maps and distance-transform-based weights are depicted with blue and red arrows, respectively. The Max Agreement Map demonstrates that most disagreements among different observers concentrate on stromal boundaries. BWFK method takes this information into account in its calculation.

The challenge in assessing inter-observer concordance for regional segmentation tasks in histopathological images primarily stems from the difficulty in delineating tissue borders. This challenge results in disagreements predominantly along the annotation boundaries, as highlighted in the *Max Agreement Map* shown in Fig 2. To mitigate this challenge, the BWFK method was introduced, utilizing distance maps derived from the annotation boundaries. The workflow of the proposed method, outlined in Fig 2, takes stromal mask images generated by observers as input, where stromal pixels are depicted in white. The processes involving the agreement maps and the creation of distance-transform-based weights are indicated by blue and red arrows, respectively.

Aggregated stromal masks are used to generate agreement maps for stromal and non-stromal prediction counts, constituting the columns in Table 1. The values in the agreement map indicate the number of observers predicting each pixel as stroma or non-stroma. To improve visibility, agreement values are color-coded, as shown in Fig 2. A Max Agreement Map is then produced by determining the pixel-wise maximum of stromal and non-stromal agreement map, highlighting the locations of disagreements.

The distance-transform-based weights are needed to decide the contribution of each pixel in the agreement maps into the kappa score. First, annotation borders are extracted from the stromal annotations, and then distance transformation is applied to calculate the distance of each pixel to the nearest boundary point. The pixel-wise mean of the distance transform maps is calculated to obtain the average distance transform map. The distance map is flattened to equalize the contribution of pixels that are further from the boundary than a certain threshold value DT, which was selected empirically as 100 pixels (See Fig 9). This boundary-based distance map is converted to a vector to obtain distance transform-based weight vector, *W* = [*w*_1_, *w*_2_,…,*w*_*M*_]. *w*_*i*_ values are normalized as

$${w\prime }_{i}=M\left(\frac{w_{i}}{\sum_{j=1}^{M}w_{j}}\right)$$ (4)

Weighted proportion of observed agreements ${\bar{P}}^{w}$, weighted proportion of agreements expected by chance ${{\bar{P}}_{e}}^{w}$ and BWFK κ^*w*^ values can be calculated as

$${\bar{P}}^{w}=\frac{1}{MN(N-1)}\left(\sum_{j=1}^{K}\sum_{i=1}^{M}{w\prime }_{i}n_{ij}^{2}-MN\right)$$ (5)

$${{\bar{P}}_{e}}^{w}=\sum_{j=1}^{N}{\left(p_{j}^{w}\right)}^{2},\;p_{j}^{w}=\frac{1}{MN}\left(\sum_{i=1}^{M}{{w\prime }_{i}n}_{ij}\right)$$ (6)

$$\kappa^{w}=\frac{{\bar{P}}^{w}-{\bar{P_{e}}}^{w}}{1-{\bar{P_{e}}}^{w}}.$$ (7)

BWFK inter-observer measurements were conducted on the clinical dataset, annotated by four pathologists to predict stromal regions. The BWFK values were compared with FK values to demonstrate the effectiveness of the proposed method. A sensitivity analysis named "shift test" was also employed to assess the effectiveness of the BWFK measure in evaluating inter-observer agreement for regional segmentation. The test aimed to quantify the impact of minor errors along annotation boundaries on the concordance between observers. In the "shift test," these small errors were simulated by randomly shifting the annotation map pixel by pixel in any direction. The rationale behind the "shift test" is rooted in the observation that inter-observer discrepancies in regional markings are predominantly noticed along the annotation boundaries.

### Detection based agreement for lymphocytes (M_L_)

A novel DBCAA was developed in this study to measure the inter-observer agreement within sets of cell predictions (See Algorithm 1). DBCAA does not need any ground truth annotations to measure inter-observer agreement, which makes it easily applicable to any inter-observer agreement assessment problem for cell detection. Besides, DBCAA can operate on the predicted center point coordinates without needing boundaries or bounding boxes of the cells.

**Algorithm 1: Distance-Based Cell Agreement Algorithm (DBCAA)**

Inputs:

     • ***N***: Number of observers

• **C** = [*c*_1_, *c*_2_,…,*c*_*N*_]: Predicted cell center point coordinate tensor, where *c*_*i*_ shows the *m*_*i*_ dimensional point array of *i*^*th*^ observer as

$c_{i}=[c_{i,1},c_{i,2},\ldots ,c_{i,m_{i}}]=[\left(x_{i,1},y_{i,1}),(x_{i,2},y_{i,2}),\ldots ,(x_{i,m_{i}},y_{i,m_{i}}\right)]$

   • ***D***_***L***_: Expected value of a lymphocyte diameter.

Output:

  • ***M***_***L***_: Cell detection agreement score among ***N*** observers

Algorithm Steps:

   1. Initialize cell agreement counter array:

*A* = []

2. For each predicted point *P* = (*x*_*i*,*k*_, *y*_*i*,*k*_) which is k^th^ point of observer i

   • Initialize agreement counter $a_{k}^{i}=0$ for point *P*

     • For each observer *j*≠*i*

   ○ Calculate nearest point distance $d_{min}^{P,j}$ from point *P* to any point in array *c*_*j*_

$d_{min}^{P,j}=\underset{i}{\min}\;d(P,c_{j,i})$

    where d(a,b)=Euclidean_Distance(a,b)

     ○ If $d_{min}^{P,j}<D_{L}$ then

      ■$a_{k}^{i}=a_{k}^{i}+1$

   • Append agreement counter value $a_{k}^{i}$ to *A*

  3. Calculate agreement score:

    $M_{L}=\frac{\sum_{i=1}^{N}\sum_{k=1}^{m_{i}}a_{k}^{i}}{N\sum_{i=1}^{N}m_{i}}$

**Fig 3:**
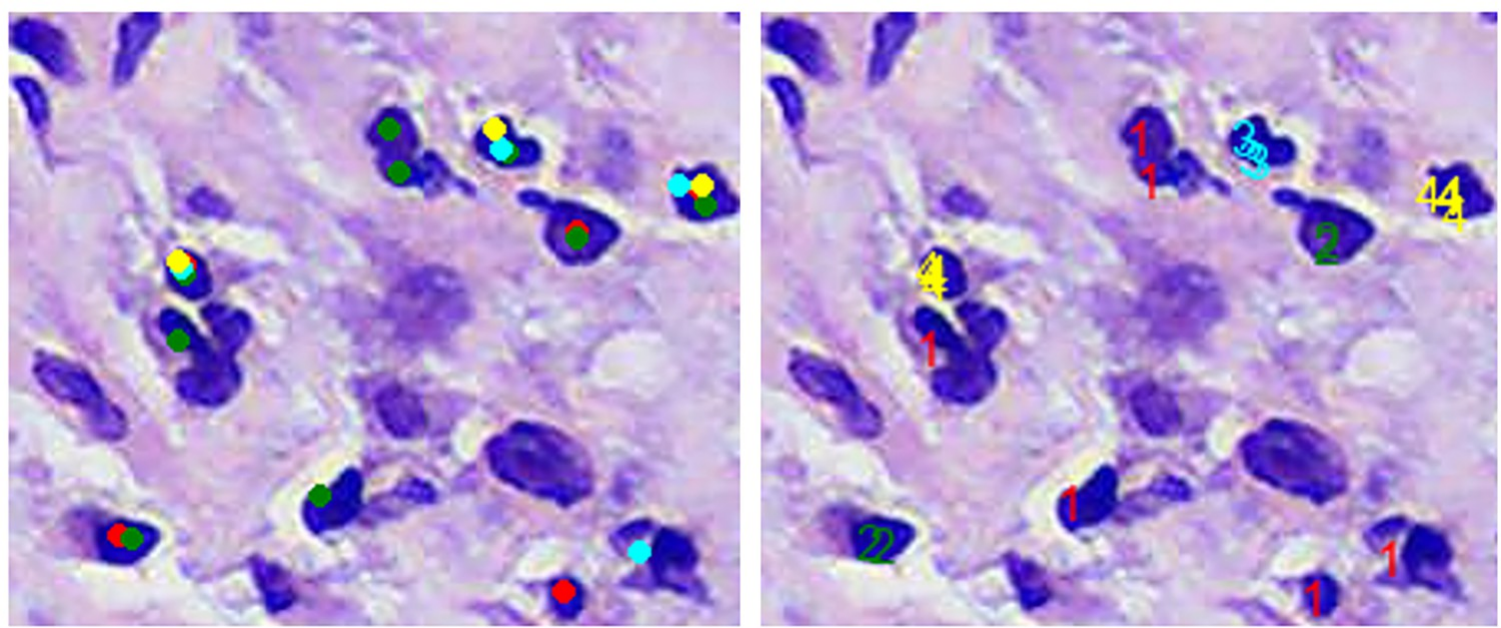
A sample demonstration of DBCAA algorithm for 4 observers. Left image: predictions of first (red), second (green), third (aqua) and fourth (yellow) observer, right image: visualization of agreement counts.

An illustration of the algorithm on a sample image is shown in Fig 3, where red, green, aqua, and yellow dots are the predictions of the first, second, third, and fourth observers, respectively, on the first image. Calculated agreement values are drawn on the second image as numbers. The cell agreement score for this sample patch image can be calculated as 55 over 84, ≅ 0.65.

The sensitivity of the DBCAA measure to changes in input point coordinates was assessed through a "shift test." In this test, annotated point coordinates were randomly altered within a specified range, defined as the maximum allowed change in cell coordinates ranging from 1 to 50 pixels. The sensitivity of the DBCAA measure to the loss of input point coordinates was evaluated through a "lost test." In this test, a certain percentage of the annotated point coordinates was randomly removed.

### Scoring based agreement for TILs scores (M_T_)

M_T_ measures to assess the degree of agreement between predicted TILs scores of different observers who are evaluating the same set of images. In this study, TILs scores were calculated from the annotations of observers. Calculating the TILs score involves assessing the percentage of stromal area occupied by lymphocytes within the tumor tissue. TILs score T can be formulated as

$$T=\frac{\sum_{j=1}^{M}A_{j}}{\sum_{x=1}^{W}\sum_{y=1}^{H}S(x,y)}$$ (8)

where *A*_*j*_ is the jth lymphocyte cell area falling into the stromal region, M is the total stromal lymphocyte cells number, W and H are the dimension of the input tissue image, *S*(*x*, *y*) is equal to 1 if (*x*, *y*) pixel point falls into stroma, equal to 0 otherwise. The average diameter of a lymphocyte was accepted as 8 μ, and the area of a lymphocyte was accepted as 50.3 μ^2^.

Several inter-observer agreement measures, such as Intra Class Correlation (ICC) Scores, Bland-Altman plots and Pearson’s Correlation Coefficients, were employed to assess M_T_ agreements.

The correlations among M_S_, M_L_, and M_T_ inter-observer agreements were also investigated, referred to as the "correlation test." To assess these correlations, the images in the clinical dataset were divided into subgroups, with ten images randomly assigned to each subgroup. This process was repeated 50 times, and Pearson correlation coefficient measurements between M_S_, M_L_, and M_T_ values were conducted for each subgroup among observers.

#### Intra Class Correlation (ICC) scores

ICC is a statistical measure widely used in research to evaluate the reliability and consistency of measurements conducted by multiple observers or methods on the same subjects [25]. It quantifies the proportion of total variability in measurements attributed to differences between subjects relative to the total variability, which includes differences within subjects. ICC values range from 0 to 1, with higher values indicating greater reliability and agreement among measurements. This versatile measure is applicable to both categorical and continuous data, including floating-point values. In scenarios involving continuous predictions, ICC becomes a valuable tool to assess the precision and consistency of measurements on a continuous scale.

#### Bland-Altman plots

Bland-Altman plots are a graphical method used to assess the agreement between two quantitative measurements or observers [26, 27]. These plots are particularly useful when comparing two different measurement techniques, instruments, or observers to identify any systematic differences, bias, or outliers between them.

Bland-Altman plots can be extended for multi-observer agreement studies to assess the agreement among multiple observers or raters. Jones et al. proposed an extension of Bland-Altman’s graphical method for assessing limits of agreement between two observers to the limits of agreement with the mean (LOAM) for multiple observers [28]. Limits of agreement, which are typically set at 1.96 times the standard deviation of the differences above and below the mean difference. These limits represent the range within which approximately 95% of the differences among observers are expected to fall, assuming the differences are normally distributed.

#### Pearson’s Correlation Coefficients

Pearson correlation coefficient is a correlation coefficient that measures linear correlation between two sets of data [29]. In this study, Pearson correlation coefficient method was employed to evaluate the correlations between M_S_, M_L_ and M_T_ agreement scores.

### Clinical dataset

**Fig 4:**
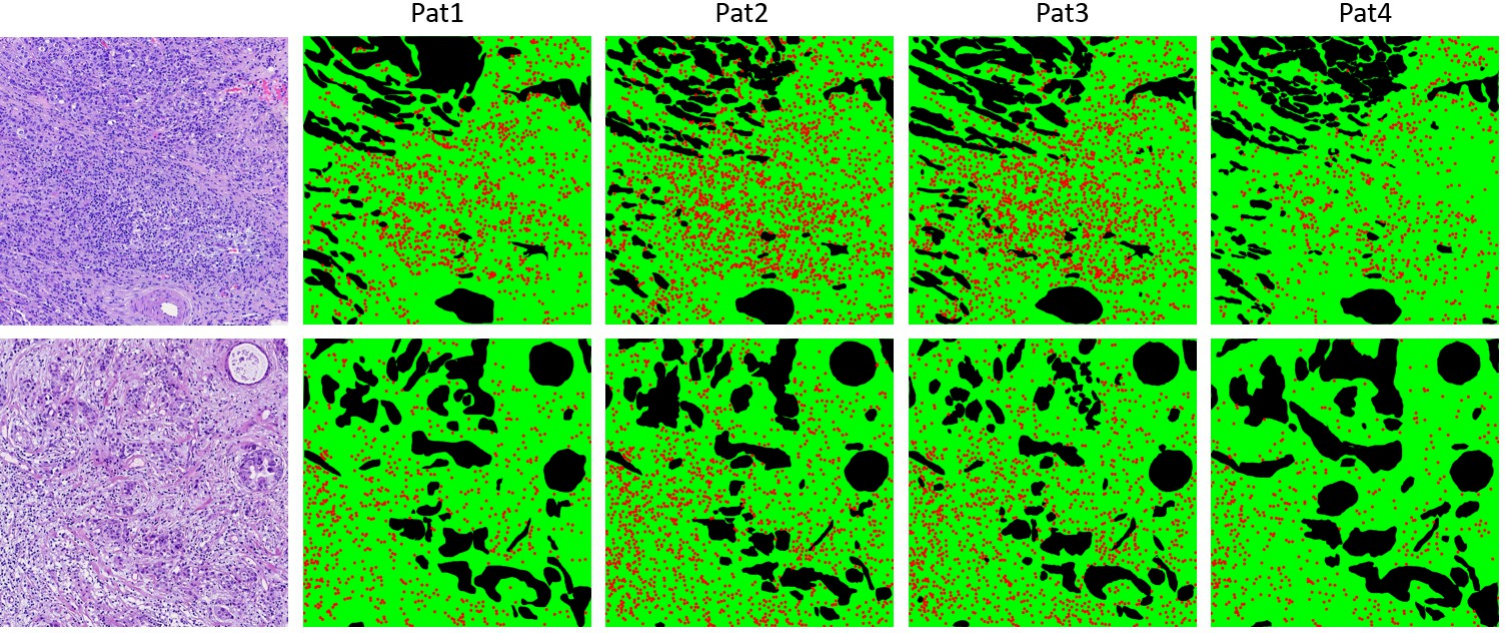
Two sample images from the clinical dataset and the pathologists’ annotation maps for stromal segmentation and lymphocyte detection. In the annotation maps, green-colored regions illustrate the stroma, black-colored regions illustrate non-stromal regions like tumor, cell debris, necrosis, fat, degenerative collagen, previous core biopsy sites, etc., and red spots show the location of lymphocyte predictions.

The clinical dataset used in the study comprised 25 H&E images from 11 patients collected at the University of Health Sciences, Department of Pathology, Haydarpaşa Numune Education and Research Hospital, Istanbul, Turkiye with due approval from the Institutional Review Board (protocol code E-62977267-771-242779795, date of approval 04.30.2024) of the hospital. The study involved female patients with an average age of 59, ranging from 28 to 79 years. All cases were diagnosed with either grade II or grade III invasive ductal carcinoma of the breast. To safeguard patient privacy, all images were subjected to a meticulous de-identification process by technicians. Personally identifiable information was removed or anonymized, ensuring confidentiality and adherence to the privacy regulations. Researchers began accessing the image data after this de-identification process on April 30, 2024. The images had a size of 3000x3000 pixels, which is approximately equal to a 0.476-mm2 area. These images were extracted from the tumor bulk region of the corresponding whole slide images to ensure that they stayed within the region where TIL scoring could be performed. Slides were scanned with the Motic EasyScan One® (Version 1.0.1.71) whole slide scanner at 40x magnification. Annotations of the clinical dataset prepared within the scope of the study were performed by four pathologists. The experiences of the participating pathologists were as follows: Pat1 (pathologist): 29 years, Pat2 (pathology resident): 2 years, Pat3 (pathology resident): 3 years, and Pat4 (pathologist): 10 years. Two sample images and their annotation maps drawn by four pathologists are illustrated in Fig 4.

### Blind annotations

**Fig 5:**
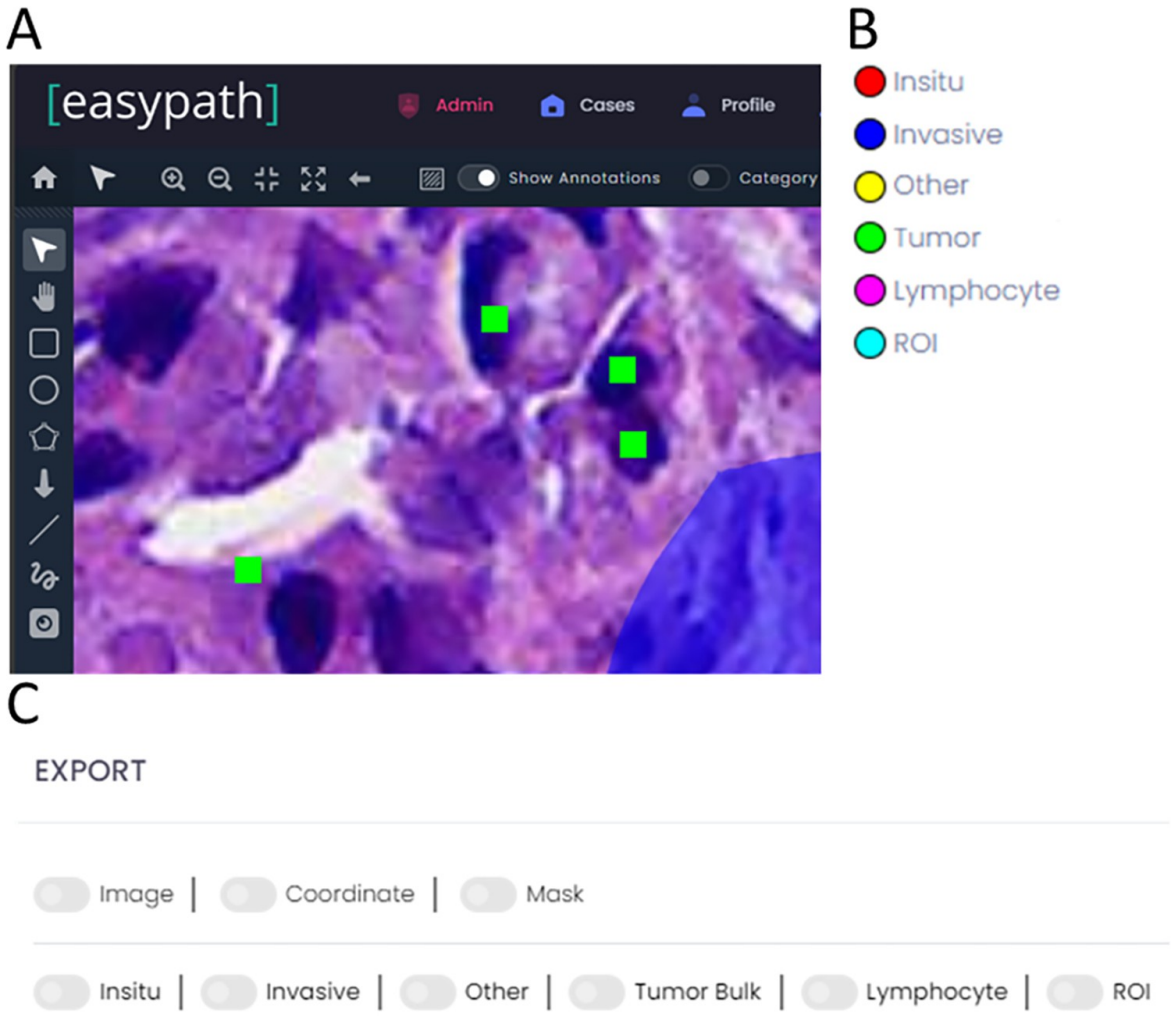
The clinical dataset and annotation tools utilized in the study. A Web based graphical user interface of the cloud platform [30] utilized for observer’s annotations blindly. B List of annotation categories of the program. C Export settings of the program for multi-observer annotations.

A web-based cloud platform [30], developed by Argenit Co. (Istanbul, Turkey), was utilized for pathologists’ annotations. The platform comprises several features that are specific to multi-observer tissue annotations (see Fig 5A).

- Different types of annotations are supported: lines, boxes, ellipses, points, polygons, and free drawings.
- Annotations can be categorized that specify the type, color, thickness, etc. of each annotation category (see Fig 5B)
- Multiple users can annotate the same image blindly.
- Annotations can be exported as ROI images, coordinates, or masks for each observer (see Fig 5C)

Cells were annotated with points, and tissue regions were outlined using a freehand drawing tool. Annotated regions were categorized as "stroma," "tumor," or "other." Regions containing cell debris, necrosis, fat, degenerative collagen, previous core biopsy sites, etc., were classified under "other" and were excluded from TILs scoring. Four pathologists performed region segmentation on a total image area of approximately 900 megapixels, equivalent to around 50 square millimeters of tissue, across the 25 tissue images in the dataset. They marked a total of 50,000 lymphocyte cells on these images.

### Validating AI in clinical practice: A pilot study

A pilot study was conducted to illustrate the validation of AI methods in clinical practice using the proposed approaches. This exemplary study emphasized validating deep learning-based cell segmentation methods, CellViT [31] and HoverNet [32], as lymphocyte detectors, and deep learning based semantic segmentation method U-Net [33] for stromal segmentation in clinical pathology settings. Pretrained versions of CellViT and HoverNet models from the publicly available datasets were employed to identify lymphocytes in our clinical image dataset. Additionally, a U-Net model was trained using the publicly available Breast Cancer Semantic Segmentation (BCSS) dataset [34] to segment stromal regions for this pilot study. The proposed DBCAA and BWFK methods were applied to evaluate the agreement between the coordinates of cells and stromal regions, as detected by the AI methods, and the annotations provided by pathologists. The consistency of TILs score assessments between the AI methods and pathologists was measured using the Intraclass Correlation Coefficient (ICC). It is important to highlight that such consensus measurements would not be feasible with existing methods in the literature due to the lack of ground truth, showcasing the clear advantage of the DBCAA and BWFK methods proposed in this study.

## Results

### M_S_: BWFK measure experiments

**Fig 6:**
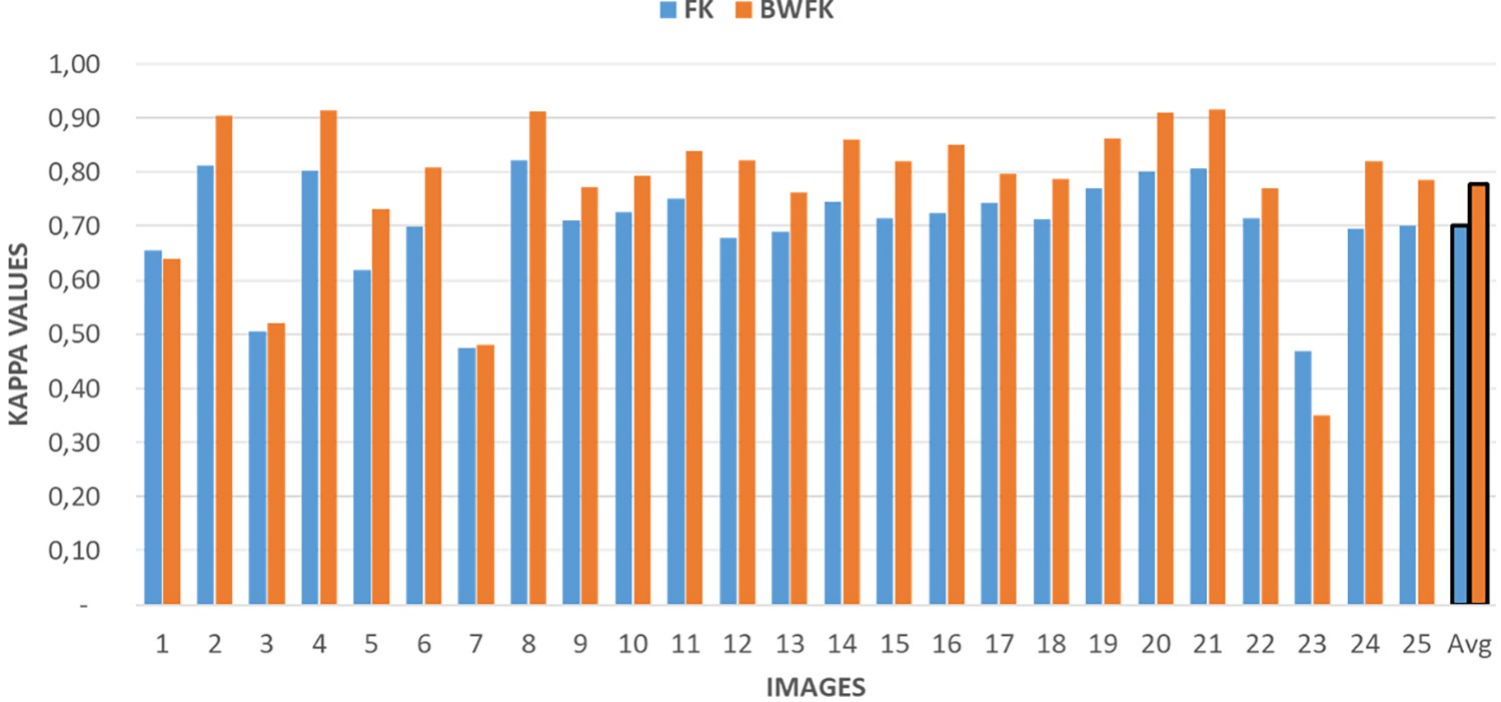
FK and BWFK agreement values among 4 pathologists on 25 images in the clinical dataset.

Comparative results between BWFK and FK methods are depicted in Fig 6. As illustrated in the figure, Kappa values were predominantly enhanced by the new BWFK method, leading to an increase in the average Kappa value from 0.70 to 0.78 (8%). These improvements stem from the ability of the BWFK method to mitigate errors occurring along the annotation boundaries (See Max Agreement Map in Fig 2).

**Fig 7:**
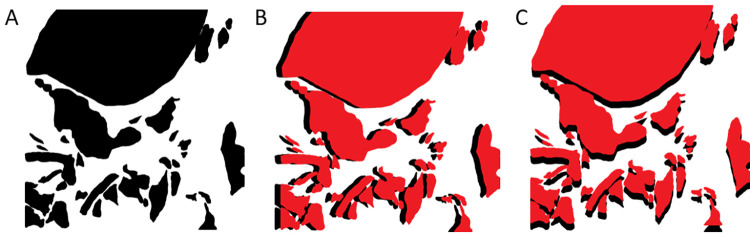
Sample images to represent the “shift test”. A: Input annotation mask image, B: Vertical shifted mask image (red) and shadowed input map (black), C: Horizontal shifted mask image (red) and shadowed input map (black).

An annotated mask image, as shown in Fig 7A, was selected from our clinical dataset to illustrate the "shift test" experiment. The input mask image was randomly shifted to simulate minor variations among observers. Fig 7B represents a right-shifted map, and Fig 7C represents an up-shifted annotation map.

**Fig 8:**
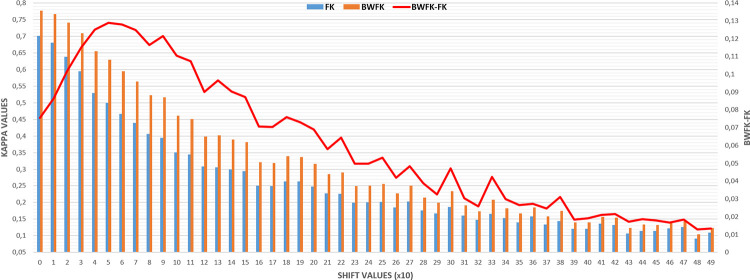
FK and BWFK values for shift values from 10 to 490 pixels. “BWFK GAIN %” percentages are also shown to represent the improvement of the proposed BWFK method against FK.

FK and BWFK measures were compared across clinical dataset images with shift values ranging from 10 to 490 pixels, as demonstrated in Fig 8. The gain percentage of the BWFK measure was calculated with 100 times the difference between BWFK and FK, divided by FK for each shift value. The results indicated that the BWFK measure exhibited greater robustness to minor disagreements along the annotation boundaries than the FK measure. The gain percentage of BWFK was 10% for original masks (0 shift) in relation to the FK value. This improvement was further increased to 16% and 19% for 20 pixels and 30 pixels shifts, respectively. The percentage gain value reached up to approximately 32% near the shift value of 100, then showed a decreasing trend. This shift value of 100 confirmed the validation of the experimentally selected DT parameter. The percentage gain values approached back to the initial value of around 10% at very high shift values.

**Fig 9:**
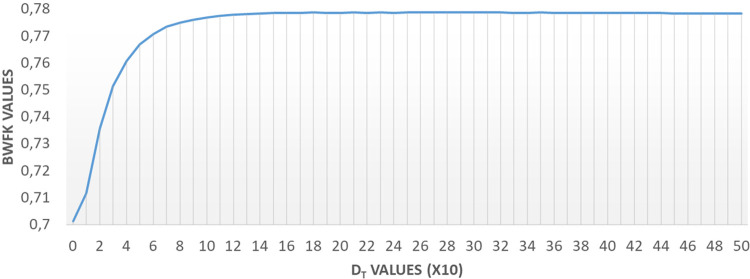
Change of BWFK values according to the DT parameter in pixels.

The BWFK method utilizes a distance threshold parameter, D_T_, to flatten the distance map. The variations in BWFK values, calculated from images in the experimental dataset, are illustrated in Fig 9 with respect to the parameter D_T_. Upon closer inspection of the figure, it becomes apparent that BWFK values experienced a significant increase up to a D_T_ of 100 pixels, after which they stabilized.

### M_L_: DBCAA measure experiments

**Fig 10:**
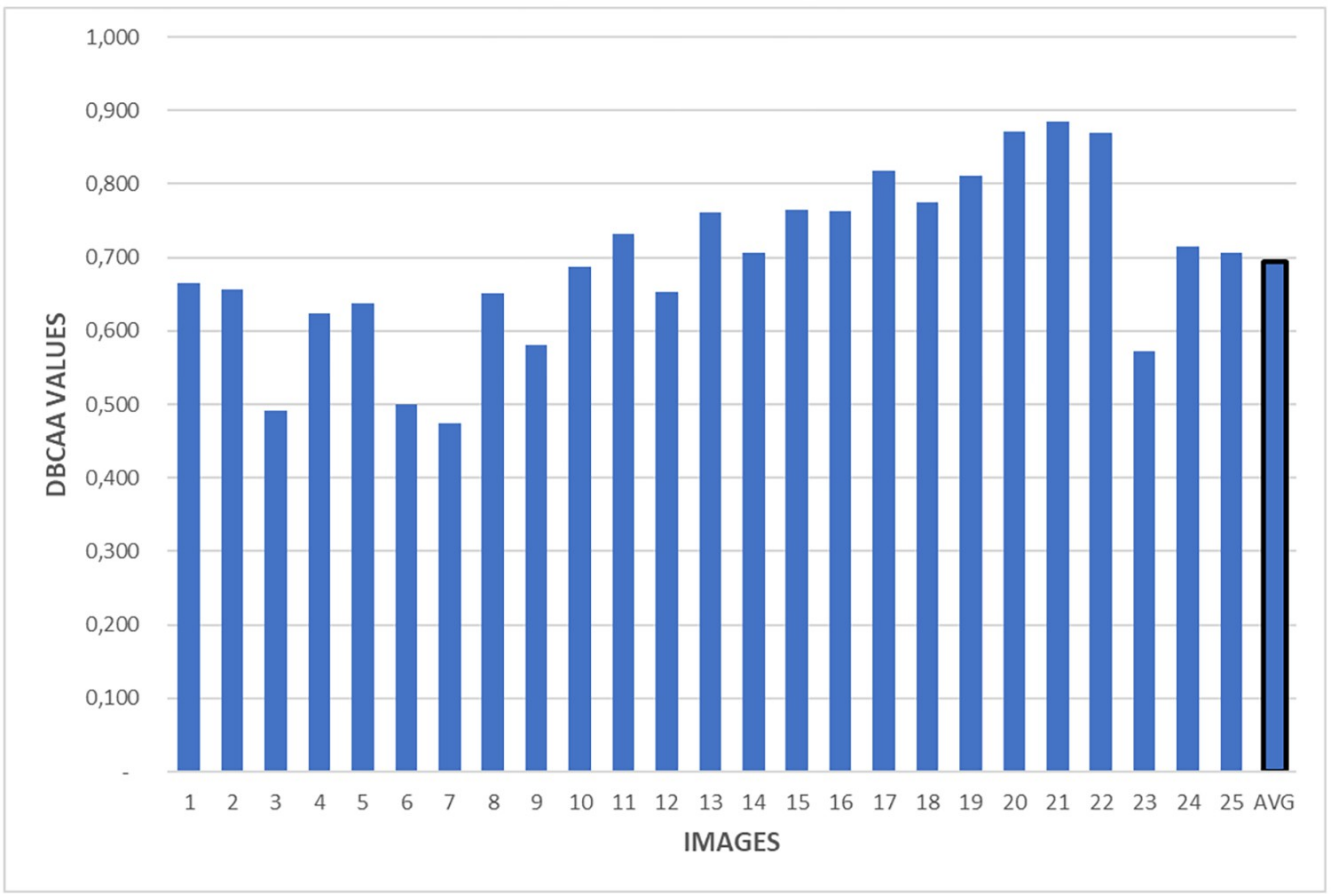
DBCAA agreement values among 4 pathologists on 25 images in clinical dataset for lymphocyte detection.

Experimental studies were carried out on lymphocyte annotations within the clinical dataset to assess the results of the DBCAA agreement. Fig 10 illustrates the DBCAA agreement values for each of the 25 images, along with the average value.

**Fig 11:**
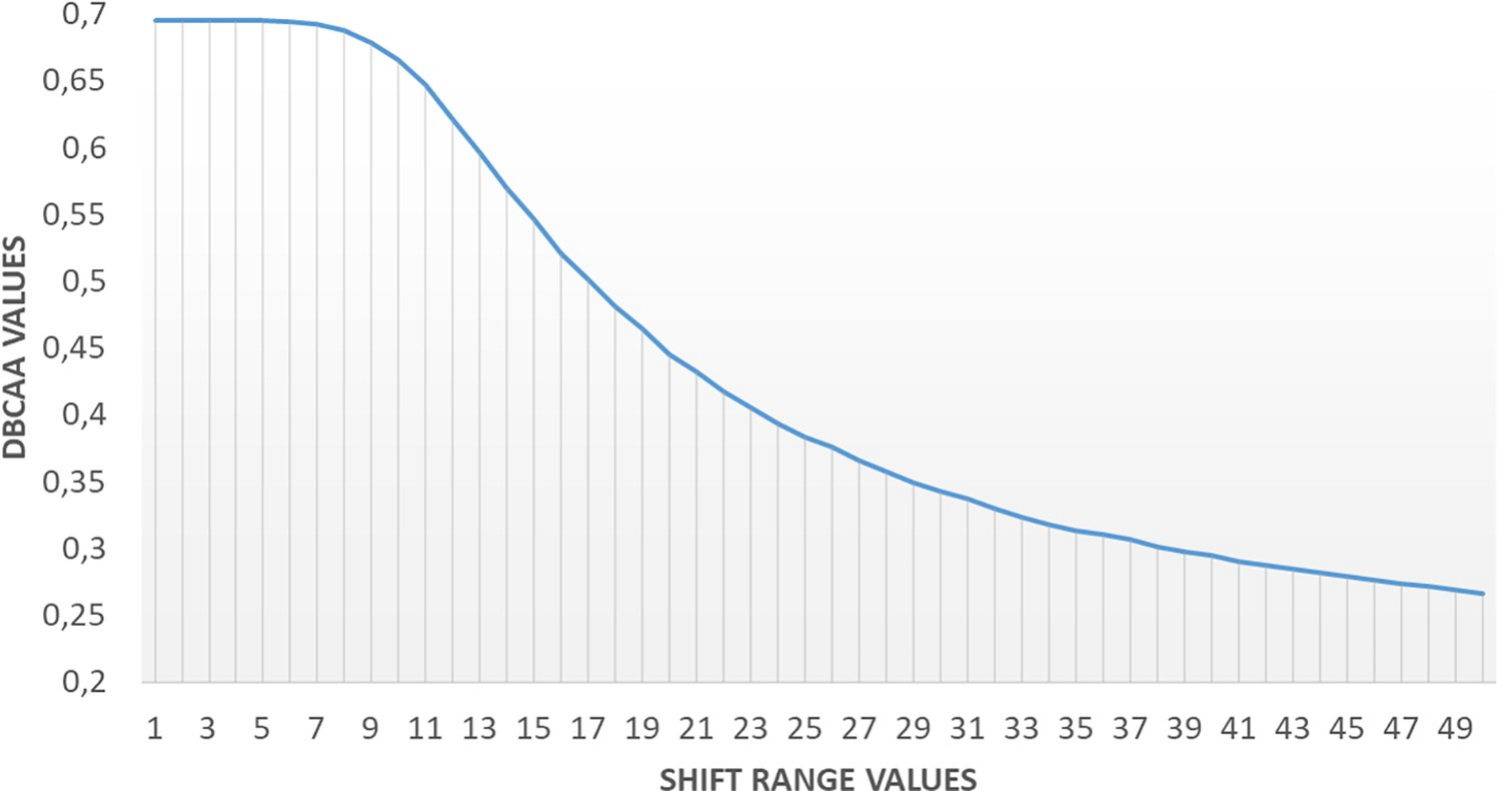
Sensitivity graph of DBCAA agreement values against point shifts.

The result of the "shift test" for DBCAA is plotted in Fig 11. As depicted in the figure, DBCAA values remained stable until the shift range value reached around 9, after which they decreased. This inflection point corresponds to the average radius of the lymphocytes in a physical sense and is linked to the DL parameter of the DBCAA algorithm.

**Fig 12:**
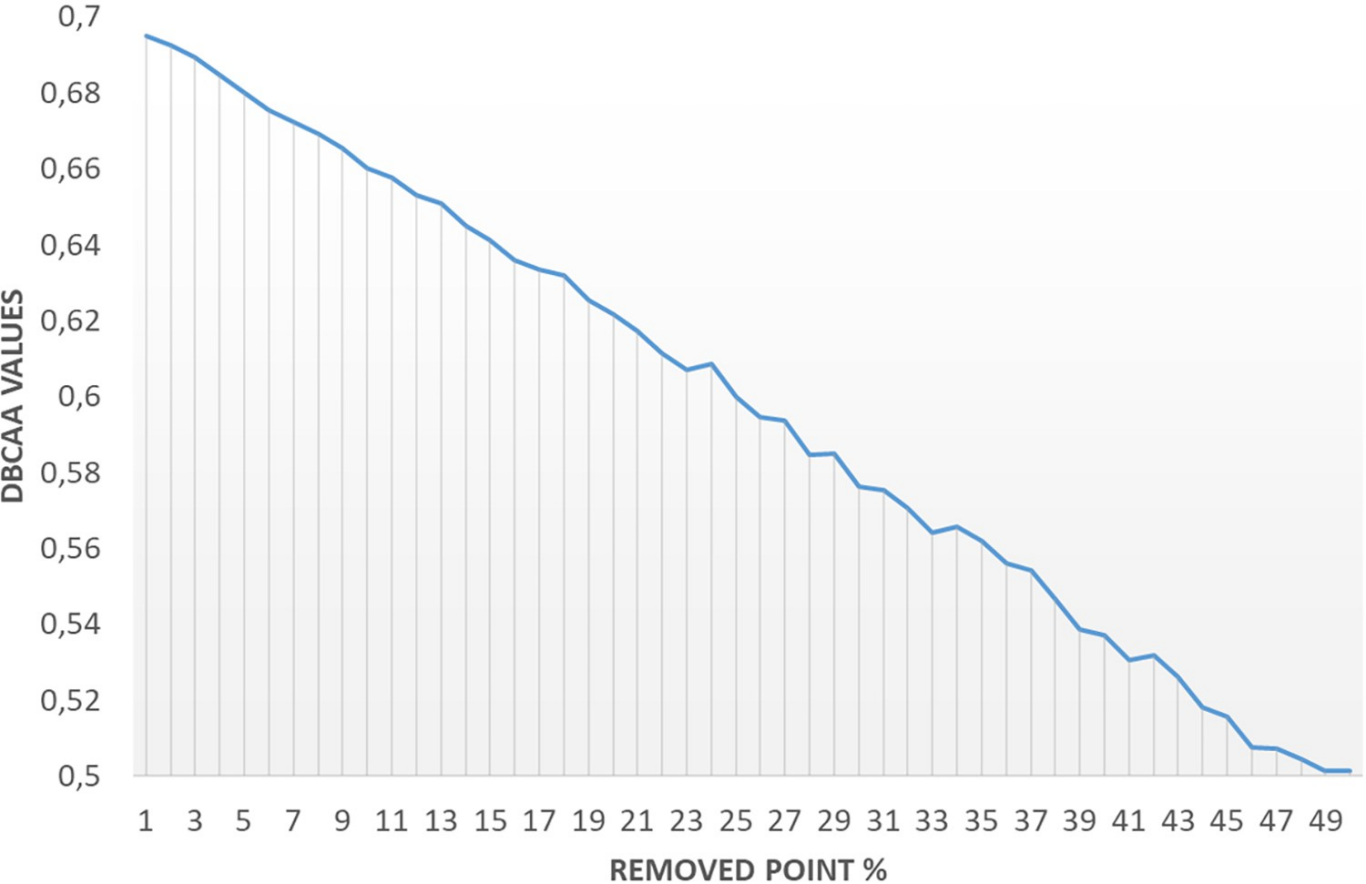
Graphical representation of sensitivity analysis for DBCAA measure values concerning the removal of lymphocyte points, ranging from 1% to 50%.

The graph illustrating the sensitivity of DBCAA measures to the loss of annotation points ("lost test") is presented in Fig 12. The results indicate that the DBCAA measure value decreased by only 0.19 (from 0.69 to 0.5) when 50% of the points were randomly removed.

### M_T_: TILs scoring experiments

**Fig 13:**
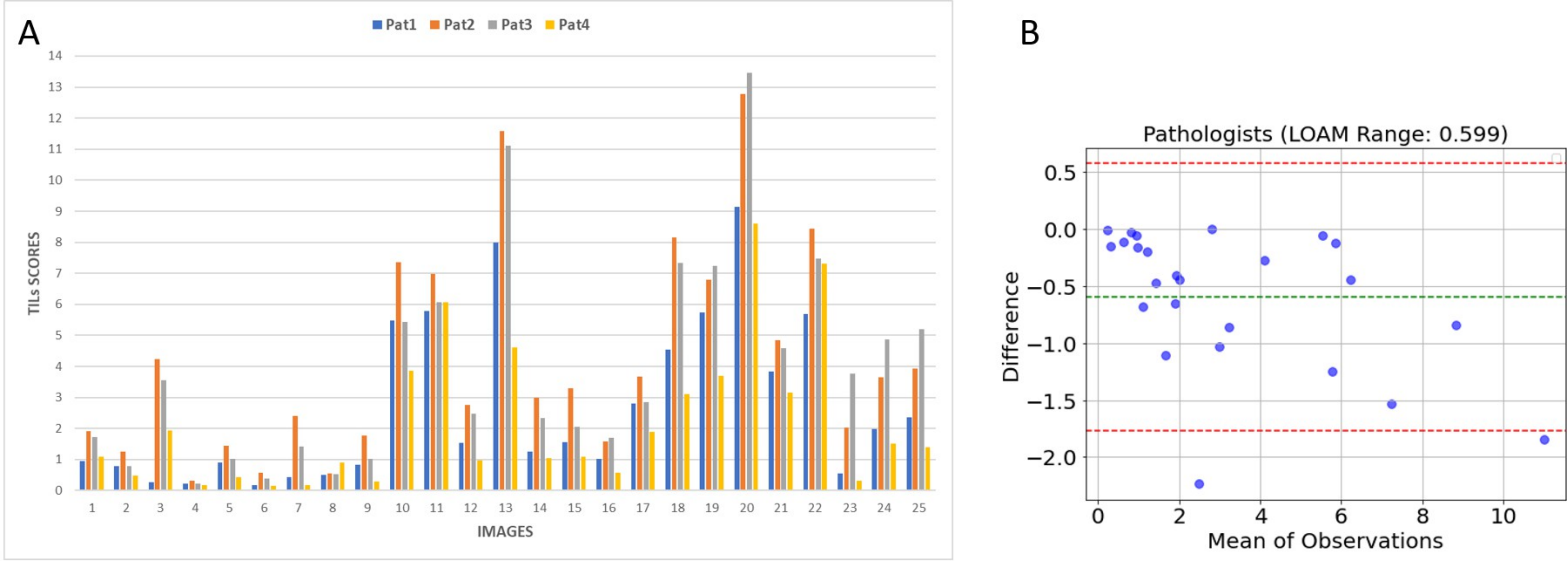
A: Calculated TIL scores for each observer and each image in the clinical dataset, B: Bland-Altman plot of the predictions. Green dotted line represents the mean difference value and two red dotted lines shows the upper and lower LOAM limits.

TILs scores were computed using stromal region and lymphocyte coordinate annotations for each image in the clinical dataset with Eq 9. The calculated TILs scores and their Bland-Altman plots are depicted in Fig 13. When examining the Bland-Altman plot, it’s noticeable that the data points were scattered around the mean line, with only two points falling outside the limits of agreement.

**Fig 14:**
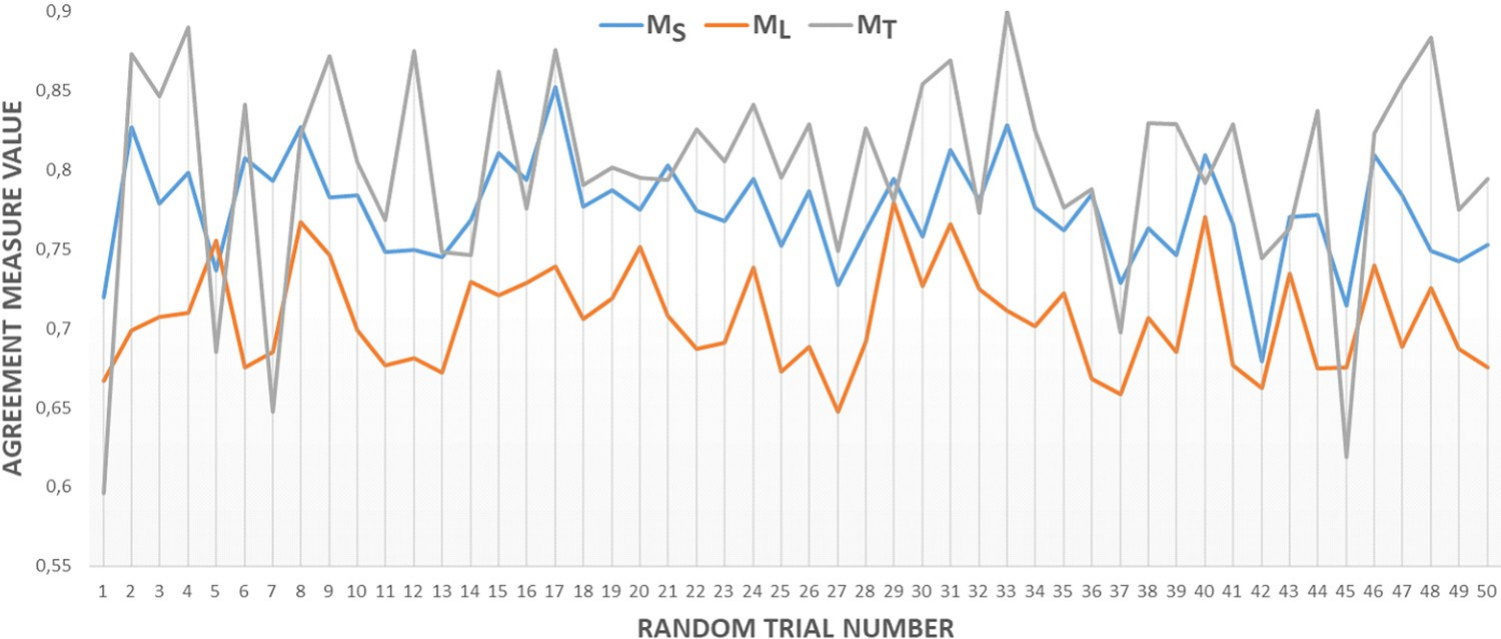
A Comparison of M_S_, M_L_, and M_T_ inter-observer agreement measures in randomly selected subsets of the experimental dataset images involved randomly selecting 10 images in each trial for analysis. Y-axis shows the value of agreement measure values of M_S_, M_L_, and M_T_ which change between 0 and 1.

The results of the “correlation test” are represented in Fig 14 and Table 2. It’s worth noting that BWKF, DBCAA, and Intra-Class Correlation (ICC) methods were utilized for M_S_, M_L_, and M_T_ measurements. The calculated Pearson correlation coefficients between M_S_, M_L_, and M_T_ measurements are presented in Table 2. The findings indicate that TILs scoring agreements (M_T_) exhibit a stronger correlation with stromal segmentation agreements (M_S_) than with lymphocyte detection agreements (M_L_).

**Table 2:** Pearson correlation coefficients of M_S_, M_L_, and M_T_ measurements represented in Fig 14.

	M_S_	M_L_	M_T_
**M**_**S**_	1	0.548	0.565
**M**_**L**_	0.548	1	0.267
**M**_**T**_	0.565	0.267	1

### Validating AI in clinical practice: A pilot study

In the pilot study, the proposed DBCAA (for lymphocyte detection) and BWFK (for stromal segmentation) methods were used to assess the agreement between cell and stromal region coordinates detected by the AI methods and the annotations provided by pathologists. The ICC method was employed to further evaluate whether the inter-observer agreement measured by DBCAA and BWFK corresponds with the final TILs score agreement. While validating the AI methods for measuring inter-observer agreement, we compared the AI-generated results with the predictions of two experienced pathologists (Pat1 and Pat4) in our observer group. This approach enabled us to more reliably compare the inter-observer agreement results produced by our methods with the final TILs scores. The results, presented in Table 3, demonstrate a high degree of consistency between the proposed inter-observer agreement measures and the final TILs scores, validating the accuracy of our methods.

**Table 3:** Comparison of the agreement measurements “M_L_ (DBCAA), M_S_ (BWFK), and M_T_ (ICC)” used in the pilot study to validate AI methods in clinical settings. The cell detection and stromal segmentation results are presented separately to highlight the specific AI methods used for prediction in the columns labeled "Cell" and "Stroma." It should be noted that for the pathologist observers, the predictors for both "Cell" and "Stroma" are identical.

Observer1		Observer2		Observer 3		M_L_ Score	M_S_ Score	M_T_ Score
Cell	Stroma	Cell	Stroma	Cell	Stroma	DBCAA	BWFK	ICC
Pat1	Pat1	Pat4	Pat4	x	x	0.785	0.792	0.903
Pat1	Pat1	Pat4	Pat4	CellViT	UNet	0.673	0.654	0.830
Pat1	Pat1	Pat4	Pat4	HoverNet	UNet	0.615	0.655	0.630

## Discussion

The study introduced a novel framework for assessing inter-observer agreement in TILs scoring on histopathological breast images. The proposed methods, namely BWFK for tissue segmentation and DBCAA for cell detection, address critical challenges in accurately measuring agreement among pathologists and AI algorithms.

To substantiate our conceptual framework, a clinical tissue image dataset was prepared from patients with invasive ductal breast carcinoma. A commercial web-based platform was chosen to facilitate the annotation process for inter-observer agreement measurements because the study team was familiar with using it. The same study can be conducted with any of the other publicly available platforms, such as Roboflow [35], Labelme [36], and Labelbox [37]. A clinical implementation guide for the proposed methods was also prepared on the Github page of [38].

In evaluating stromal segmentation, BWFK demonstrated superior performance compared to traditional FK, particularly along the tissue boundaries. The "shift test" sensitivity analysis highlighted BWFK’s robustness to minor errors along annotation boundaries, with BWFK showing a remarkable improvement of up to 32% over the traditional FK method. This underscores its effectiveness in mitigating inter-observer discrepancies.

The performance of the method was further characterized by the distance threshold parameter (DT), which exhibited stability and improvement up to a threshold of 100.

For lymphocyte detection, DBCAA presents a groundbreaking advancement by eliminating the need for ground truth annotations. The method exhibited stability with minimal sensitivity to changes in point coordinates and proved resilient to the loss of annotation points. These characteristics make DBCAA a versatile and practical tool for inter-observer agreement assessment in cell detection, overcoming challenges associated with obtaining ground truth data.

A pilot study was conducted to validate AI-based cell detection and stromal segmentation methods in clinical practice using the DBCAA and BWFK methods. The results indicate that the consensus among two experienced pathologists was higher than that between the pathologists and any of the AI methods reviewed in this experiment. Additionally, the findings demonstrate that the proposed DBCAA and BWFK inter-observer agreement measurements are consistent with the final TILs score measurements, confirming their applicability for validating AI methods in clinical settings. While the current study focuses primarily on measuring inter-observer agreement, the strong correlation between these agreement scores and the quantification of TILs suggests promising potential for future applications in improving outcome predictions, such as patient survival or treatment response.

The study also highlighted the correlation between stromal segmentation (M_S_) and TILs score predictions (M_T_), tackling the origin of high inter-observer variability in TILs scoring. The proposed framework contributes to resolving the elevated inter-observer variability in breast cancer pathology by providing standardized and interpretable methods. These methods can be seamlessly integrated into a variety of histopathological applications.

The field of breast cancer diagnosis is currently undergoing dynamic development, with methodologies and standards continuously evolving. In this context, our study aimed to provide valuable insights and practical tools to inform and support ongoing advancements in Tumor-Infiltrating Lymphocytes (TILs) assessment. By adhering to the guidelines established by the International Immuno-Oncology Biomarker Working Group on Breast Cancer, our research not only highlights the importance of TILs scoring in current research paradigms but also demonstrates how our novel methodologies—BWFK and DBCAA—can enhance the accuracy, efficiency, and reproducibility of such assessments. It’s essential to note that while our study primarily focused on TILs scoring to showcase the potential of our methodologies, their applicability extends beyond this specific use case. Our approach was designed to be adaptable to a wide range of histopathological analyses, providing valuable tools to the pathology community as it navigates the complexities of integrating new biomarkers into clinical practice.

There are several limitations to address in our study. Our primary objective was to demonstrate the potential and adaptability of these methods in a clinical setting, focusing on the TILs scoring task as a proof-of-principle study. While our dataset was smaller in scale compared to typical clinical research studies, it provided sufficient detail for a preliminary demonstration of the effectiveness of BWFK and DBCAA in addressing real-world pathology-related problems. The limited size of the dataset can also be attributed to the significant time investment needed for image annotation. This is evident from the extensive effort required by four pathologists to annotate a total of 50,000 cells and mark 900 mega-pixel areas across the 25 images in the dataset. Our findings offer valuable preliminary evidence of the methods’ applicability and set a foundation for future research, which we plan to undertake with larger and more diverse datasets to further evaluate and refine our approaches. Our study’s approach also has limitations in controlling for observer bias and differences in annotation precision, which are inherent challenges in histopathological evaluations. Additionally, the proof-of-principle nature of our framework, while innovative, means that our findings must be interpreted with caution as they may not yet be generalizable to broader clinical practices without further validations. Moreover, our current analysis did not account for the full spectrum of TILs variability across different types of breast cancer, which could have affected the overall adaptability of the methods. These aspects highlight the necessity for follow-up studies to assess the robustness and reliability of BWFK and DBCAA methods under various clinical and pathological conditions.

## Conclusions

This proof-of-principle research presents a comprehensive and innovative approach to evaluating inter-observer agreement in TILs scoring on histopathological breast images. The proposed BWFK for stromal segmentation and DBCAA for cell detection contribute valuable tools for pathologists and AI algorithms alike. The study’s contributions extend beyond TILs scoring, offering adaptable solutions for broader histopathological image analysis.

The framework’s validation on a clinical dataset demonstrated the effectiveness of BWFK and DBCAA in enhancing inter-observer agreement assessments. These methods show promise in addressing the limitations associated with traditional measures, particularly in the context of tissue segmentation and cell detection, where boundaries and ground truth annotations present challenges. The BWFK method enhances the standard FK method by up to 32% by reducing inter-observer discrepancies along regional boundaries. Additionally, a pilot study was designed to illustrate the process of validating AI methods using the DBCAA and BWFK methods in clinical settings.

As the field of histopathological image analysis increasingly incorporates AI algorithms, the proposed framework provides a crucial bridge for assessing the concordance between AI predictions and human observations. The outcomes of the study lay the foundation for standardized, reliable, and interpretable inter-observer agreement assessments in breast cancer pathology, with implications for improved diagnostics and treatment strategies. Future work may involve further validation on diverse datasets and exploration of additional applications across various histopathological analyses, addressing the size limitation of our experimental cohort in this study and applying it in broader clinical practices with the full spectrum of TIL variability across different types of breast cancer.
